# BIOFRAG – a new database for analyzing BIOdiversity responses to forest FRAGmentation

**DOI:** 10.1002/ece3.1036

**Published:** 2014-03-27

**Authors:** Marion Pfeifer, Veronique Lefebvre, Toby A Gardner, Victor Arroyo-Rodriguez, Lander Baeten, Cristina Banks-Leite, Jos Barlow, Matthew G Betts, Joerg Brunet, Alexis Cerezo, Laura M Cisneros, Stuart Collard, Neil D'Cruze, Catarina da Silva Motta, Stephanie Duguay, Hilde Eggermont, Felix Eigenbrod, Adam S Hadley, Thor R Hanson, Joseph E Hawes, Tamara Heartsill Scalley, Brian T Klingbeil, Annette Kolb, Urs Kormann, Sunil Kumar, Thibault Lachat, Poppy Lakeman Fraser, Victoria Lantschner, William F Laurance, Inara R Leal, Luc Lens, Charles J Marsh, Guido F Medina-Rangel, Stephanie Melles, Dirk Mezger, Johan A Oldekop, William L Overal, Charlotte Owen, Carlos A Peres, Ben Phalan, Anna M Pidgeon, Oriana Pilia, Hugh P Possingham, Max L Possingham, Dinarzarde C Raheem, Danilo B Ribeiro, Jose D Ribeiro Neto, W Douglas Robinson, Richard Robinson, Trina Rytwinski, Christoph Scherber, Eleanor M Slade, Eduardo Somarriba, Philip C Stouffer, Matthew J Struebig, Jason M Tylianakis, Teja Tscharntke, Andrew J Tyre, Jose N Urbina Cardona, Heraldo L Vasconcelos, Oliver Wearn, Konstans Wells, Michael R Willig, Eric Wood, Richard P Young, Andrew V Bradley, Robert M Ewers

**Affiliations:** 1 Department of Life Sciences, Imperial College LondonSilwood Park Campus, Ascot, SL5 7PY, U.K; 2 Stockholm Environment InstituteStockholm, Sweden; 3 Centro de Investigaciones en Ecosistemas, Universidad Nacional Autónoma de México (UNAM)Morelia, Mexico; 4 Department of Forest & Water Management, Ghent UniversityGhent, Belgium; 5 Lancaster Environment Centre, Lancaster UniversityLancaster, U.K; 6 Department of Forest Ecosystems and Society, Oregon State UniversityCorvallis, Oregon; 7 Southern Swedish Forest Research Centre, Swedish University of Agricultural SciencesAlnarp, Sweden; 8 Departmento de Métodos Cuantitativos y Sistemas de Información, Universidad de Buenos AiresBuenos Aires, Argentina; 9 Department of Ecology and Evolutionary Biology, University of ConnecticutStorrs, Connecticut; 10 Center for Environmental Sciences and Engineering, University of ConnecticutStorrs, Connecticut; 11 Nature Conservation Society of South AustraliaAdelaide, SA, Australia; 12 The World Society for the Protection of AnimalsLondon, U.K; 13 Departamento de Entomologia, Instituto Nacional de Pesquisas da Amazônia (INPA)Manaus, Brazil; 14 Geomatics and Landscape Ecology Research Laboratory, Carleton UniversityOttawa, ON, Canada; 15 Terrestrial Ecology Unit, Ghent UniversityGhent, Belgium; 16 Centre for Biological Sciences, University of SouthamptonSouthampton, U.K; 17 351 False Bay Drive, Friday Harbor, Washington, 98250; 18 School of Environmental Sciences, University of East AngliaNorwich, U.K; 19 International Institute of Tropical Forestry, USDA Forestry ServiceRio Piedras, Puerto Rico; 20 Institute of Ecology, FB2, University of BremenBremen, Germany; 21 Agroecology, Department of Crop Sciences, Goettingen UniversityGoettingen, Germany; 22 Natural Resource Ecology Laboratory, Colorado State UniversityFort Collins, Colorado; 23 Swiss Federal Institute for Forest Snow and Landscape Research WSLBirmensdorf, Switzerland; 24 OPAL, Imperial College LondonLondon, U.K; 25 INTA EEA, Bariloche – CONICETBariloche, Argentina; 26 Centre for Tropical Environmental and Sustainability Science and School of Marine and Tropical Biology, James Cook UniversityCairns, Qld, Australia; 27 Departamento de Botânica, Universidade Federal de PernambucoRecife, Brazil; 28 Faculty of Biological Sciences, University of LeedsLeeds, U.K; 29 Instituto de Ciencias Naturales – ICN, National University of ColombiaBogotá, Colombia; 30 Department of Ecology and Evolutionary Biology, University of TorontoToronto, ON, Canada; 31 Department of Zoology, Field Museum of Natural HistoryChicago, Illinois; 32 Sheffield Institute for International Development, University of SheffieldSheffield, U.K; 33 Departamento de Entomologia, Museu Paraense Emílio Goeldi (MPEG)Belém, Brazil; 34 Department of Forest and Wildlife Ecology, University of Wisconsin-MadisonMadison, Wisconsin; 35 The University of QueenslandBrisbane, Qld, Australia; 36 10 River St Marden, Marden, 5070, Australia; 37 Royal Belgian Institute of Natural SciencesBrussels, Belgium; 38 Life Sciences Department, The Natural History MuseumLondon, U.K; 39 Centro de Ciências Biológicas e da Saúde, Universidade Federal de Mato Grosso do SulCampo Grande, Brazil; 40 Department of Fisheries and Wildlife, Oregon State UniversityCorvallis, Oregon; 41 Department of Parks and Wildlife, Manjimup Research CentreManjimup, WA, Australia; 42 Department of Biology, Carleton UniversityOttawa, ON, Canada; 43 Department of Zoology, University of OxfordOxford, U.K; 44 Centro Agronómico Tropical de Investigación y Enseñanza (CATIE)Turrialba, Costa Rica; 45 School of Renewable Natural Resources, Louisiana State University Agricultural CenterBaton Rouge, Louisiana; 46 BDFFP, INPAManaus, Brazil; 47 Durrell Institute of Conservation and Ecology, School of Anthropology and Conservation, University of KentCanterbury, U.K; 48 School of Biological Sciences, University of CanterburyCanterbury, New Zealand; 49 School of Natural Resources, University of Nebraska-LincolnLincoln, Nebraska; 50 Ecology and Territory Department, School of Rural and Environmental Studies, Pontificia Universidad JaverianaBogotá, Colombia; 51 Instituto de Biologia, Universidade Federal de UberlândiaMinas Gerais, Brazil; 52 Institute of Zoology, Zoological Society of LondonLondon, U.K; 53 The Environment Institute, School of Earth and Environmental Sciences, The University of AdelaideAdelaide, SA, Australia; 54 Durrell Wildlife Conservation TrustTrinity, Jersey, U.K

**Keywords:** Bioinformatics, data sharing, database, edge effects, forest fragmentation, global change, landscape metrics, matrix contrast, species turnover

## Abstract

Habitat fragmentation studies have produced complex results that are challenging to synthesize. Inconsistencies among studies may result from variation in the choice of landscape metrics and response variables, which is often compounded by a lack of key statistical or methodological information. Collating primary datasets on biodiversity responses to fragmentation in a consistent and flexible database permits simple data retrieval for subsequent analyses. We present a relational database that links such field data to taxonomic nomenclature, spatial and temporal plot attributes, and environmental characteristics. Field assessments include measurements of the response(s) (e.g., presence, abundance, ground cover) of one or more species linked to plots in fragments within a partially forested landscape. The database currently holds 9830 unique species recorded in plots of 58 unique landscapes in six of eight realms: mammals 315, birds 1286, herptiles 460, insects 4521, spiders 204, other arthropods 85, gastropods 70, annelids 8, platyhelminthes 4, Onychophora 2, vascular plants 2112, nonvascular plants and lichens 320, and fungi 449. Three landscapes were sampled as long-term time series (>10 years). Seven hundred and eleven species are found in two or more landscapes. Consolidating the substantial amount of primary data available on biodiversity responses to fragmentation in the context of land-use change and natural disturbances is an essential part of understanding the effects of increasing anthropogenic pressures on land. The consistent format of this database facilitates testing of generalizations concerning biologic responses to fragmentation across diverse systems and taxa. It also allows the re-examination of existing datasets with alternative landscape metrics and robust statistical methods, for example, helping to address pseudo-replication problems. The database can thus help researchers in producing broad syntheses of the effects of land use. The database is dynamic and inclusive, and contributions from individual and large-scale data-collection efforts are welcome.

## Introduction

The conversion and resulting fragmentation of native habitat are frequently implicated as primary causes of terrestrial biodiversity loss (Fahrig 2003; Gardner et al. 2009; Krauss et al. 2010). Consequently, there has been a growing and widespread scientific interest on understanding biologic responses to fragmentation as one aspect of land-use change, with a large number of reviews (Niemelä 2001; Chalfoun et al. 2002; Fahrig 2003; Cushman 2006; Ewers and Didham 2006a; Nichols et al. 2007; Prugh et al. 2008; Arroyo-Rodriguez and Dias 2010; Didham 2010; Hadley and Betts 2012; Tscharntke et al. 2012). Despite such interest, studies have produced a very complex set of results that are challenging to synthesize in a meaningful way. A major challenge for synthesis across studies of biodiversity responses to habitat and landscape change, including fragmentation, lies in dealing with studies that differ fundamentally in experimental design and methods (Chalfoun et al. 2002; Arroyo-Rodriguez and Dias 2010), measure the fragmentation process in different ways (Fahrig 2003), and investigate diverse – often interconnected – drivers and response variables (McGarigal and McComb 1995; Ewers and Didham 2006b, 2007; Fletcher et al. 2007). Published papers show little consensus on which aspects of individual (e.g., growth, abundance) and community level (e.g., richness, *β*-diversity) responses or aspects of landscape structure and composition (e.g., patch size, shape, edges, and total landscape characteristics such as habitat amount) should be studied (Fahrig 2003; Ewers and Didham 2006a; Lindenmayer and Fischer 2007; Ewers et al. 2010; Melles et al. 2012). This lack of consensus has added to the confusion regarding biodiversity responses to fragmentation rather than facilitating comparisons between studies for meaningful answers that can inform conservation action and management.

The lack of uniformity is exemplified in the diverse literature on edge effects (Murcia 1995; Ries et al. 2004; Ewers and Didham 2006a,b). Edges or boundaries between habitat patches alter species interactions (Fagan et al. 1999), the trophic structure of communities (Laurance et al. 2011), mortality (Laurance et al. 2006), and flows of individuals and resources through landscapes (Huxel and McCann 1998). The patch-mosaic model (Forman 1995), whilst widely used, does not take account of the high levels of environmental heterogeneity that characterize modified landscapes (McGarigal and Cushman 2002). The separation of the fragmented landscape into habitat interior, edge, and matrix is often arbitrary (Laurance et al. 1998). However, this separation should be determined from species' response functions to the edge (Ewers and Didham 2006a,b; Ewers et al. 2007), which depend on species' functional traits (Ryall and Fahrig 2006) and vary with edge type (Restrepo et al. 1999), patch quality (Magrach et al. 2011), patch connectivity, and matrix quality. Moreover, previous research has often ignored asymmetric impacts of boundaries across patches (Ewers and Didham 2006b; Fonseca and Joner 2007), the cumulative effect of multiple edges (Malcolm 1994; Fletcher 2005), “matrix contrast” (Ries et al. 2004; Reino et al. 2009; Prevedello and Vieira 2010), and matrix impacts on patch connectedness (Bender and Fahrig 2005; Watling et al. 2011). Relative effects of area and edge are difficult to isolate from each other (Fletcher et al. 2007), and patch isolation may be best interpreted as a measure of habitat amount rather than landscape configuration per se (Bender et al. 2003; Fahrig 2003).

Confusion over suitable approaches for quantifying both various aspects of landscapes and biodiversity responses has led, inevitably, to contradictory and/or inconsistent results. The complex nature of biodiversity, coupled with a fundamental lack of data, exacerbates the problem (Gardner et al. 2007; Gardner 2012). Systematic analyses of multitaxa responses to experimentally created landscapes of forest fragments are rare (Margules 1992; Bierregaard et al. 2001; Barlow et al. 2007; Ewers et al. 2011; Laurance et al. 2011). Traditional meta-analysis of published results is hampered, as studies often fail to include complete descriptions of study sites (Harper et al. 2005), omit statistical information such as standard errors (Chalfoun et al. 2002), are based on different data types and qualities, or use differing sampling methods and efforts across sites and “treatments” (Gardner et al. 2007; Nichols et al. 2007), frequently preventing determination of effect sizes (Prevedello and Vieira 2010).

We have generated a database to overcome some of above-mentioned difficulties. The relational BIOFRAG database compiles primary biodiversity datasets from fragmented landscapes around the world. The current focus is on biodiversity response to forest fragmentation reflecting the interests of the principal investigators. However, the database itself could be extended to include other types of land cover and land cover change processes. Data can be queried, for example to extract studies that measured the same response variable for a specified taxonomic group, thereby increasing sample size and reducing geographic bias. Consistent techniques, such as connected component labeling used in FRAGSTAT (McGarigal et al. 2002) or improved fragment delineation (Lefebvre et al. 2013), can be used to characterize fragmentation descriptors across landscapes coherently based on the geo-location of sampling plots combined with high spatial resolution maps, which are becoming increasingly available at global scales (Hansen et al. 2013; Sexton et al. 2013). Subsequently, rigorous analyses based on a variety of metrics can be applied to this set of uniform predictor and response variables. The database stores relevant data to conduct analyses in a standardized way. It can aid in raising awareness about additional information needed for answering specific questions, and about under-sampled regions and taxonomic groups. Through interstudy comparisons, it can pave the way for the design of standardized, taxon-specific methods to measure responses to fragmentation.

This article describes (1) the organization of data for the BIOFRAG database, its structure and current status, (2) how the datasets may be used to analyze habitat fragmentation impacts consistently across landscapes and taxa, and (3) minimum data requirements and processing steps required to add further inventories to the database. We encourage forest fragmentation researchers to share their data to further expand the database in an attempt to close data gaps and to address problems of study and geographic bias. The project's website (http://biofrag.wordpress.com/) provides background knowledge, information on contributing researchers, and will feature future publications associated with the database.

## Data Compilation and Preprocessing

Compilation of the database began by first identifying suitable data via literature search (including the terms “fragmentation” and “forest” and “species abundance” and “biodiversity”) and contacting corresponding authors, discussions with presenters at conferences, and a metadata search of the PREDICTS (Projecting Response of Ecological Diversity in Changing Terrestrial Systems) database (Newbold et al. 2012). Certain essential criteria had to be met before inclusion of a dataset from these sources (see Table S1). The dataset had to contain quantitative and therefore analyzable data for responses of species. The dataset measured species responses in plots or along transect located within different habitat fragments. The dataset contained GPS coordinates, time stamps, and land cover information for plots or transects sampled. If plots were measured repeatedly, the study had to specify whether data were stored separately for each sampling period or whether aggregation techniques were applied to the response variables.

**Figure 1:**
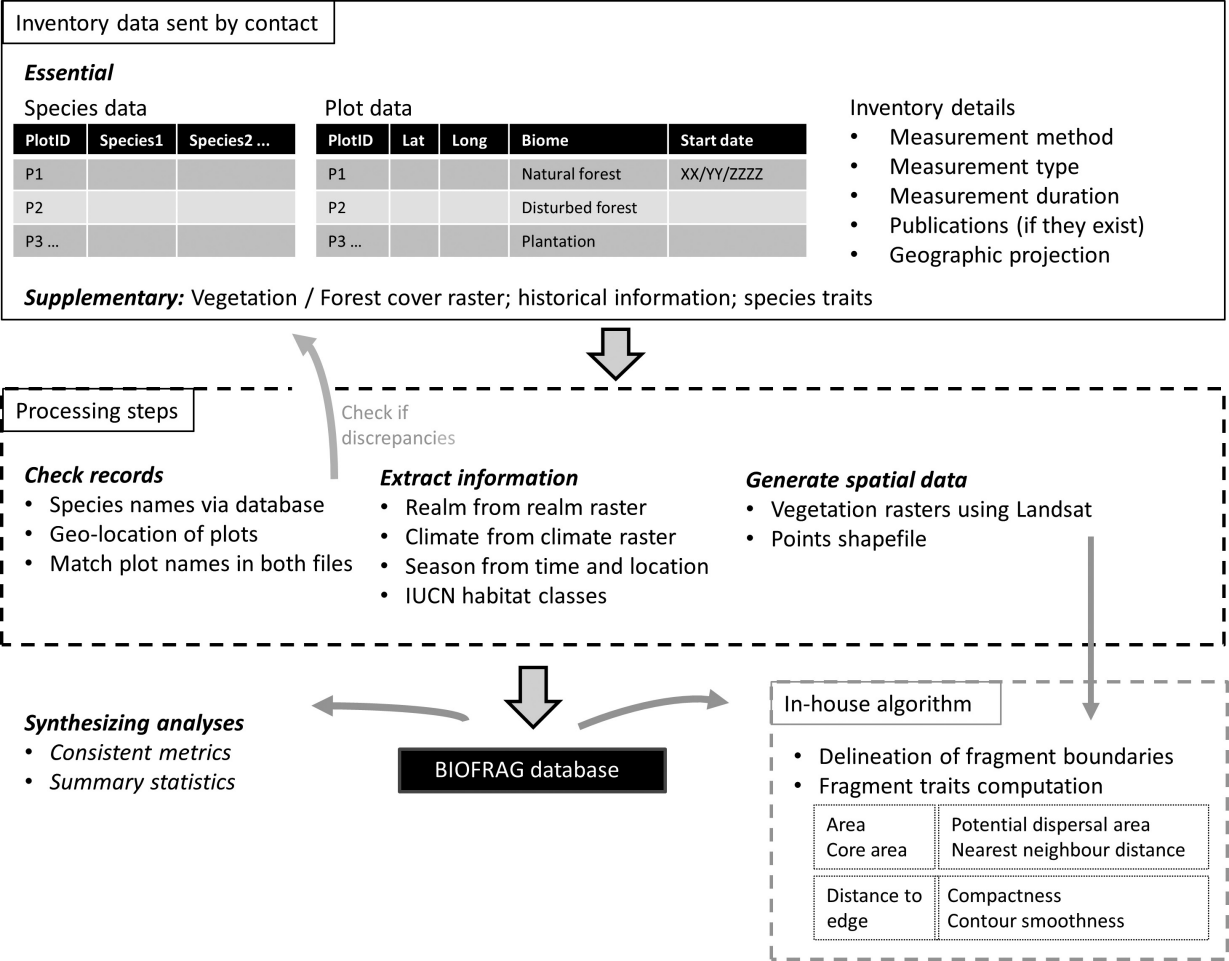
Preprocessing steps carried out before adding new inventories to the relational BIOFRAG database. Binary habitat maps are further processed using an in-house fragment delineation and characterization algorithm that generates maps of fragments and attribute tables for each fragment ID (e.g., patch area, length of edge, core area, patch connectedness).

All datasets underwent a series of preprocessing steps (Fig. 1; Table S2 in Supporting Information). These steps will be applied whenever a new dataset is added to the BIOFRAG database. The steps relevant for checking species data are: (1) checking species names against the “Catalogue of Life” (http://www.catalogueoflife.org/annual-checklist/2013/) or in case of birds against the global bird database (http://avibase.bsc-eoc.org/avibase). If species names are not found, they are checked against additional databases (http://amphibiaweb.org/; http://reptile-database.org/). If required, we reference back to the authors of the study for clarification. The classification will be updated regularly to account for changes in species names or taxonomic groupings.

For each inventory, we list months during which data were recorded at a given location and identify the corresponding season. Vegetation types (for plots in fragments and in differing matrix types) were reclassified into one of the IUCN categories from the vegetation type provided for that plot. IUCN classes include, beside natural vegetation types, six land-use categories (i.e., arable land, pasture, plantations, rural gardens, urban areas, and tropical/subtropical heavily degraded former forest).

**Figure 2:**
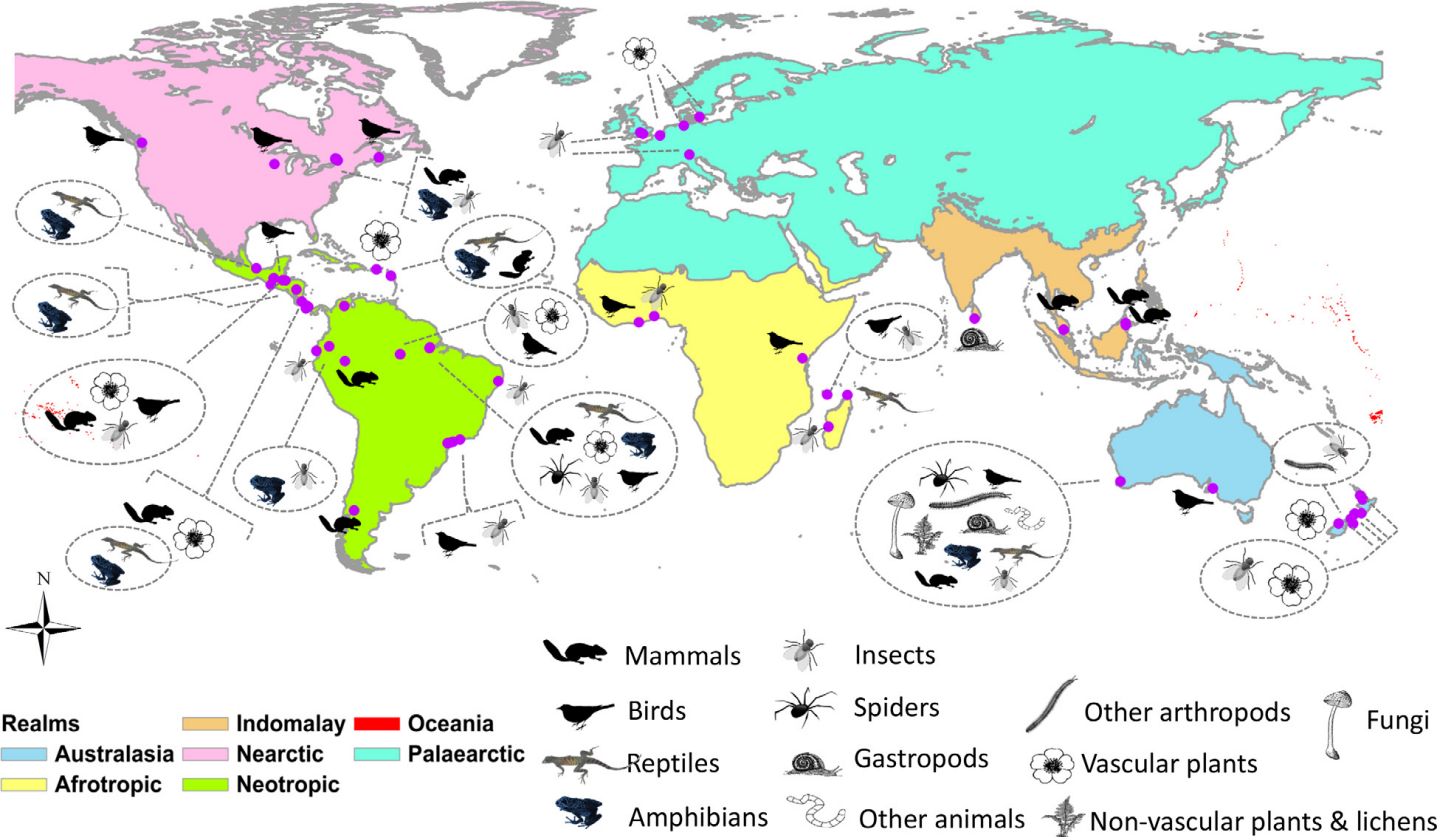
Geographical coverage of current BIOFRAG datasets. All landscapes are shown on a base map of the WWF's biogeographic realms.

The geo-locations of sample points were used to generate spatial data and checked against a global map of country and land cover for validation and subsequent reference back to the author in case of inconsistencies. For each inventory, a feature file (points) is generated and used to create minimum convex polygons for each inventory (center coordinates displayed on Fig. 2). These are used to locate suitable Landsat imagery using USGS Earth Explorer (in time as close as possible to the date of the field measurements) covering an area of at least + 5 km distance outside the polygon's boundaries. We will check whether the landscape is covered by recently generated, multitemporal high-resolution forest maps (Hansen et al. 2013) and includes those in the database. Most of the Landsat images that we will use will contain reflectance data at a spatial resolution of 30 m pixels. The images will be corrected (radiometric and atmospheric corrections) and used to generate binary maps (MapFile.TIFF) of vegetation cover (forest/nonforest), maps that additionally map disturbed/forest regrowth and more detailed maps if ground measurements allow. Simple forest cover statistics will be extracted from the maps, which are then also stored in the database. If the polygon of an inventory intersects with a large forest patch, the area mapped will exceed the + 5 km distance threshold. Maps will be validated by comparing to MODIS (Friedl et al. 2010) land cover maps and local maps if provided. We extracted the inventory's location with regard to biodiversity hotspots and protected areas (IUCN & UNEP-WCMC 2010) using maps downloaded from http://protectedplanet.net/ (accessed 26 September 2013) and from http://www.conservation.org/where/priority_areas/hotspots/, accessed 26 September 2013.

## Features of the Database

Datasets in the BIOFRAG database may contain measurements of response variables at different levels of ecological detail (i.e., presence or absence of species vs. abundance, relative abundance or percentage coverage). Measurements may reflect the response of single species (e.g., variation in population traits) or communities (e.g., community composition) that have been measured once (as temporal snapshots) or repeatedly (as time series).

The database (status February 3rd, 2014) currently holds single- and multiple-species inventories collected from 58 fragmented forest landscapes worldwide (Table 1). It encompasses 9830 unique species, most of them from the Neotropic and Australasia realms (Table 2). The 58 landscapes are distributed across six of the eight World Wildlife Fund (WWF) biogeographic realms (Fig. 2, Table 1) and nine of the 14 WWF biomes (Table 2). Species-rich “tropical and subtropical moist broadleaf forests” are represented by 20 landscapes in the Neotropic realm, three landscapes in the Afrotropic realm, and four in the IndoMalay realm. “Temperate broadleaf and mixed forests” are represented by eight landscapes in the Nearctic realm, five landscapes each in the Palaearctic and Australasia realms, and one landscape in the Neotropic realm (Table 2). Few landscapes are located in other forest biomes, for example, “tropical and subtropical dry broadleaf forests”.

**Table 1:** Unique species sampled per taxonomic group (S) in each WWF realm. Realms include the Afrotropic (AT), Neotropic (NT), IndoMalay (IM), Australasia (AA), Nearctic (NA), and Palaearctic (PA) realms. Because some species have been recorded in more than one realm, the numbers will not sum to 9830 (= number of unique species across all landscapes). LS –Number of landscapes sampled for a particular taxonomic group. Status February 3rd 2014. Insects include ants, bees, and orchid bees (Hymenoptera), beetles (Coleoptera), blowflies, and fruitflies (Diptera), bugs (Heteroptera), butterflies and moths (Lepidoptera), caddisflies (Trichoptera), cicadas (Hemiptera), cockroaches and termites (Blattodea), dobsonflies (Megaloptera), dragonflies and damselflies (Odonata), earwigs (Dermaptera), grasshoppers and crickets (Orthoptera), mantises (Mantodea), mayflies (Ephemeroptera), net-winged insects (Neuroptera), scorpionflies (Mecoptera), stick insects (Phasmatodea), and stoneflies (Plecoptera)

	AT	NT	IM	AA	NA	PA	S	LS
Mammals	0	187	113	11	6	0	234	12
Birds	252	733	0	192	132	0	1286	16
Amphibians	0	226	0	8	9	0	150	13
Reptiles	11	205	0	0	0	0	217	10
Insects	421	1597	0	2315	51	137	4007	20
Chilopoda	7	0	0	22	0	0	29	2
Diplopoda	27	0	0	7	0	0	34	2
Isopoda	14	0	0	8	0	0	22	2
Spiders	0	116	0	88	0	0	204	2
Gastropods	0	0	65	5	0	0	70	2
Annelids	0	0	0	8	0	0	8	1
Platyhelminthes	0	0	0	4	0	0	4	1
Onychophora	0	0	0	2	0	0	2	1
Vascular plants	0	1003	0	680	0	434	1900	15
Nonvascular plants and lichens	0	0	0	320	0	0	320	1
Fungi	0	0	0	449	0	0	449	1
Number of landscapes	6	24	4	9	9	6		

**Table 2:** Number of landscapes sampled in World Wildlife Fund (WWF) biomes and WWF realm (see Table 1 for abbreviations)

	AT	NT	IM	AA	NA	PA
Deserts and Xeric Shrublands	1	–	–	–	–	–
Mediterranean Forests, Woodlands, and Scrub	–	–	–	2	–	–
Montane Grasslands and Shrublands	–	–	–	1	–	–
Temperate Broadleaf and Mixed Forests	–	1	–	5	8	5
Temperate Coniferous Forests	–	–	–	–	1	1
Temperate Grasslands, Savannas, and Shrublands	–	–	–	1	–	–
Tropical and Subtropical Dry Broadleaf Forests	1	3	–	–	–	–
Tropical and Subtropical Grasslands, Savannas, and Shrublands	1	–	–	–	–	–
Tropical and Subtropical Moist Broadleaf Forests	3	20	4	–	–	–

AT, Afrotropic; NT, Neotropic; IM, IndoMalay; AA, Australasia; NA, Nearctic; PA, Palaearctic.

Insects are the dominant species group in the database, the majority of them being classified only to the level of morpho-species (Table 1). Birds are relatively better represented than other taxonomic groups confirming expectations (e.g., Gardner et al. 2007). Based on estimates of vertebrate species richness given in the biodiversity chapter of the Millennium Ecosystem Assessment (Hassan et al. 2005), our database currently holds 19% of bird species recorded for the Neotropic and Nearctic realms, and more than 11% of all Afrotropical and Australasian birds species. The database holds 15% and 12% of mammals recorded for Neotropic and IndoMalay realms, but <2% of mammals recorded for the Nearctic and Australasia realms. The database covers <5% of the amphibian species in the Neartic and Australasia realms, but 8% of those in the Neotropics; it also includes 8.0% and 0.6% of reptile species described for the Neotropic and Afrotropic realms, respectively.

The aim of the database is to assess biologic responses to habitat fragmentation rather than provide a complete collection of species records on the globe. Gap analysis, however, does highlight some of the major data gaps. Addressing the lack of data for indicator groups such as mammals and amphibians in the “subtropical/tropical moist forests” biome of the Afrotropic and IndoMalay realms, for example, could be prioritized in future collation efforts, given the importance of these biome–realms (Giam et al. 2012) and their vulnerability to habitat loss and degradation (Malhi et al. 2008; Ewers et al. 2011). Although, invertebrate taxa are most critically undersampled and understood.

Measuring temporal trends in biodiversity responses to fragmentation can provide insights into patterns of species loss and community disassembly, for example, as shown by the long-term Biological Dynamics of Forest Fragments project (BDFFP) (Laurance et al. 2011). The database contains three long-term time series of data: the tree inventories carried out at the BDFFP since 1980 (Laurance et al. 2011) and at the Luquillo Experimental Forest since 1988 (Heartsill Scalley et al. 2010) and the annual bird surveys in South Australia's Mount Lofty Ranges (since 1999). Most inventories, however, are single snapshots in time, and some have been carried out over different vegetative seasons.

**Figure 3:**
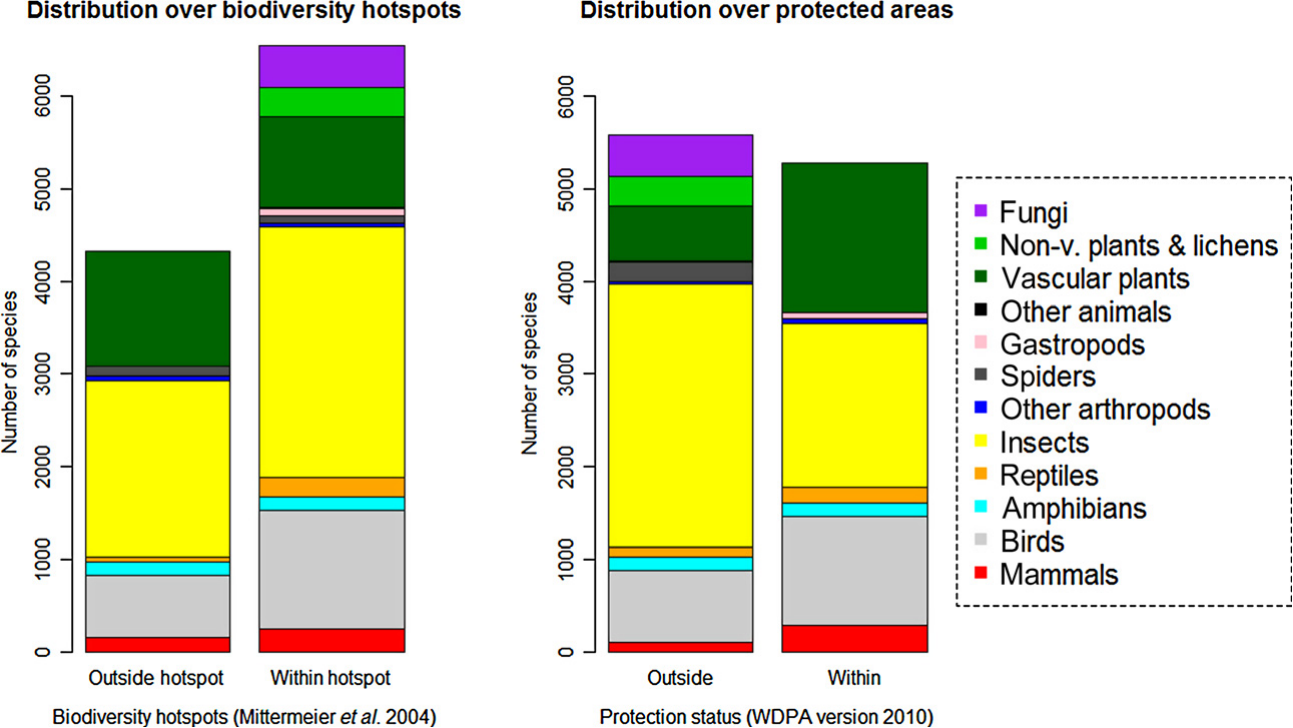
Distribution of unique species in the 58 landscapes across biodiversity hotspots and protected areas.

Comparing the response of single species to fragmentation in different landscapes (geographically clearly separated, i.e., distances between them significantly exceed distances among plots within landscapes) may allow conclusions on the generality of the response to fragmentation descriptors such as patch size, edge, and patch connectedness in the context of other factors that may influence the response (e.g., variation in abiotic environments, disturbance regimes, and matrix structure). The database currently holds records for 711 species whose response to fragmentation had been sampled in multiple landscapes. However, some taxonomic groups (e.g., birds and amphibians) are represented better than others (Table 3). Sixty-four percent of the landscapes (and 60% of species) in the database are from a total of 13 biodiversity hotspots, which themselves represent only 2.5% of the global land area (Mittermeier et al. 2011). Birds, herptiles, and insects have been more sampled within biodiversity hotspots than elsewhere (Fig. 3). Gastropoda, other invertebrate groups (Annelida, Platyhelminthes, Onychophora), nonvascular plants, lichens, and fungi were sampled exclusively from biodiversity hotspots, although there is a general lack of data for these groups. The majority of sampled landscapes (72%) include plots from within protected areas; they encompass 49% of unique species in the database. Vascular plants, birds, herptiles, and mammals have been more sampled within protected areas (Fig. 3).

**Table 3:** Number of species sampled in more than one partially forested landscape in each taxonomic group. The database currently holds 445 species recorded in exactly two landscapes, 202 species in exactly three landscapes, 35 species in four landscapes, and 20 species in five landscapes. Four bird species that occur widespread in the Neotropics have been sampled in six landscapes (*Coereba flaveola, Cyclarhis gujanensis, Pachyramphus polychopterus, Trogon rufus*). Three bird species (*Dryocopus lineatus, Piaya cayana, and Xenops minutus*), widely distributed widely distributed in the Neotropics, and one amphibian (*Rhinella marina*) have been in sampled in seven landscapes and one bird species (*Vireo olivaceus*) in eight landscapes

	Mammals	Birds	Amphibians	Reptiles	Insects	Vascular plants
2 Landscapes	48	188	24	17	75	93
3 Landscapes	13	140	10	9	1	29
4 Landscapes	–	27	1	4	–	3
5 Landscapes	–	15	–	1	3	1
6 Landscapes	–	4	–	–	–	–
7 Landscapes	–	3	1	–	–	–
8 Landscapes	–	1	–	–	–	–

## Structure of the Database

The database was designed following normalization rules to minimize redundancy and dependency and to isolate data. This means that design changes (i.e., additions and modifications of a field) can be made in just one table, which then propagate through the rest of the database (Codd 1971). Thereby, data are addressed by value rather than position and larger tables are divided into smaller ones with relationships defined among them.

**Figure 4:**
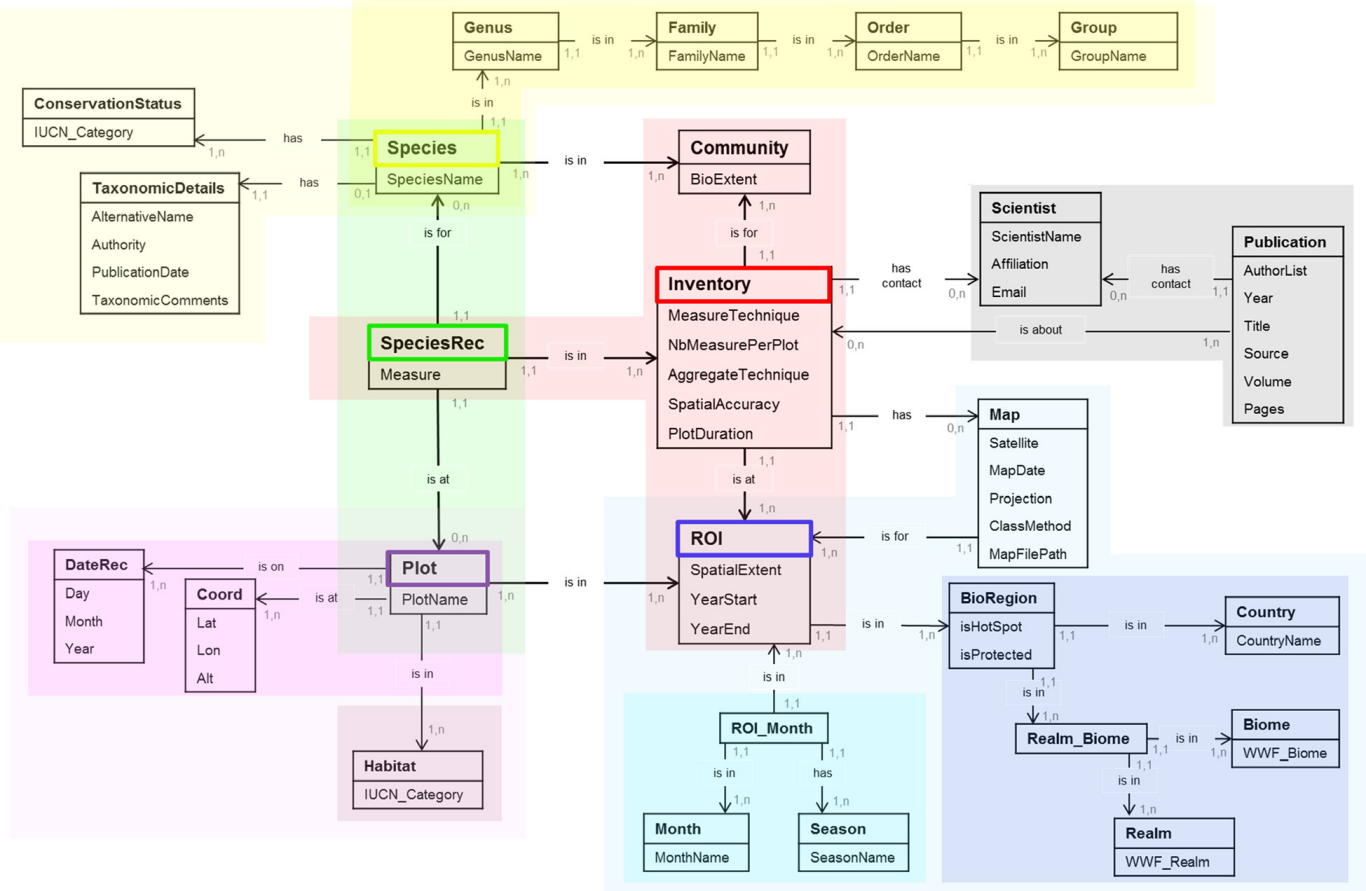
The conceptual model of the database describes tables (header = table name), their attributes (rows in the table), and the logical relationships between tables. The notation A (1,1) — is in → (1,n) B is a one-to-many relationship (“There is one and only one A in B. B has ≥1 of A”). The database also contains one-to-one and many-to-many relationships. Colors in the graph represent the five main groups of associations in the database. For example, purple: *BIOREGION* is an association of country, biome, and realm, and it relates to a region of interest, *ROI*; blue: *ROI_MONTH* is an association of months and season and pertains to a *ROI*. COMMUNITY does not have specific associations yet.

The standardization of data derives from the constraints of the fixed architecture of the database. The database is designed around a circular (and fixed) relation with six central tables (see Fig. S1): *SPECIES*, *PLOT*, *SPECIESREC*, *INVENTORY, COMMUNITY*, and *ROI* (Fig. 4: conceptual model; Fig. S2: structure of the database). Three extra tables define entries in *SPECIES* and *PLOT*. Further tables provide essential information for queries and analyses but not for the functioning of the database. *SPECIES* holds names of species recorded in at least one landscape and links to species taxonomy via tables *GENUS*, *FAMILY*, *ORDER,* and *GROUP*. Two extra tables define currently accepted names and synonyms as additional entries (*TAXONOMICDETAILS*) and IUCN protection status (*CONSERVATIONSTATUS*) for each species. A pair of coordinates (stored in *COORD*) at a unique time point (stored in *DATEREC*) is a plot stored in the *PLOT* table. Each plot contains information on whether it is located within or outside a protected area (*isProtected*). Each plot's IUCN habitat is stored in *HABITAT*. This allows change in habitat over time, for example, in time series of inventories. *SPECIESREC* links plots, species, and inventory data and also stores information on the response variable measured (*Measure*). An *INVENTORY* is a collection of measurements on a set of species (*COMMUNITY*) and a set of plots (region of interest, ROI) (Fig. 4, Fig. S2). Each *INVENTORY* entry provides information on the method (*MeasureTechnique*: e.g., pitfall traps) used to measure a response variable, the number of measures (e.g., pitfall traps at a time) per plot (*NBMeasurePerPlot*), whether measures have been summed or averaged within a plot (*AggregateTechnique*), accuracy of spatial locations (*SpatialAccuracy*), and the time period over which the data were collected (*PlotDuration*). Thus, one inventory can store measurements taken in the same plots at different times, provided that authors and publications are identical.

Each ROI is a set of plots, and plots (coordinates + date) can serve in several ROIs (stored in *ROI*) (Fig. 4, Fig. S2). Additional links allow the extraction of background information via association tables (a table with two foreign keys). For example, *REALM_BIOME* links each inventory to a biogeographic realm (*REALM*) and the predominant habitat type (*BIOME*), both as defined by WWF (Olson and Dinerstein 2002). BIOREGION contains one field indicating whether an ROI is located within or outside a biodiversity hotspot (*isHotSpot*), whilst linking to tables *COUNTRY* and *REALM_BIOME*. *ROI_MONTH* links to seasonal information (*SEASON*) for a specific month of recording in the field (*MONTH*) at a given location. A scientist stored in the table *SCIENTIST* (ScientistName, Affiliation, and Email address) is a contact for one or more publications stored in *PUBLICATION*. One publication (e.g., journal article, report) can describe one or more inventories, and one inventory may be described in several or zero publications (Fig. 4, Fig. S2).

## Data Access, Queries, and Research Opportunities

Data stored in the BIOFRAG database are available for noncommercial scientific use, but researchers have to request access to individual datasets from the dataset authors. We are currently developing a routine that will allow a freely available meta-data search on all datasets to identify their suitability in the context of specific research questions posed by interested researchers. Researchers wishing to contribute to the database are asked to contact the principal investigators of the project (S1: m.pfeifer@imperial.ac.uk; r.ewers@imperial.ac.uk) as automatic uploading of datasets is not yet implemented.

The database will enable consistent analyses of fragmentation impacts on biodiversity that can help account for recent advances in the spatial analyses of landscape traits (Wagner and Fortin 2005; Vogt et al. 2009; Larsen et al. 2012; Lefebvre et al. 2013) and of species' responses to fragmentation (Westphal et al. 2003; Driscoll and Weir 2005; Betts et al. 2006, 2007; Ewers and Didham 2006a, 2007, 2008; Ewers et al. 2009; Laurance et al. 2011; Didham et al. 2012). Existing and new metrics quantifying the responses must be able to address challenges of intercorrelation between predictors and spatial scaling, for example, by using remotely sensed data to characterize landscape attributes at different spatial scales (Prugh 2009; Eigenbrod et al. 2011). These metrics should also help to tackle the problem of pseudo-replication, for example, by accounting for turnover-by-distance relationships and environmental gradients driving background variation in biodiversity (Eigenbrod et al. 2011; Ramage et al. 2012). Derived standardized results can then be more effectively synthesized, for example via meta-analyses.

Structure Query Language (SQL) queries can be designed by research teams depending on the analyses and meta-analytical reviews they want to apply, for example, to re-examine previous hypotheses (Table 4) or answer questions such as “Is there a critical patch size in the stepping stone model for a given group of species?” and “How do habitat and biogeographic affinities of species determine their response to forest fragmentation?”, which can help in identifying ways for managing the ability of the matrix to mediate the biodiversity impacts of habitat loss and fragmentation. Finding answers to questions such as “What are the key functional groups for detecting and monitoring the effects of forest fragmentation on the provision of essential ecosystem services?” and “How many and which species are lost and gained in fragments over time?” can aid in assessing the biodiversity value of fragments in the context of their respective landscapes, which is relevant to inform conservation policies and the design of sustainable landscapes and the design of sustainable landscapes (Westphal et al. 2007). SQL queries can also be applied to derive database statistics (e.g., number of species per taxonomic group/biome/realm, number of datasets with repeated measurements) and to identify data gaps, highlighting areas in need of further research and data collection.

**Table 4:** Selected research questions that could be asked when studying biologic responses to habitat fragmentation

	Raised by
Questions on functional responses	
Does the degree of pollination specialization control susceptibility of trees to fragmentation?	Prevedello and Vieira (2010)
Does dispersal mediate impact of fragmentation on demography of forest-dependent species?	Lampila et al. (2005), Slade et al. (2013)
Do species show threshold responses to habitat configuration following fragmentation?	Villard et al. (1999), Ewers and Didham (2006a)
Does the relative impact of fragmentation versus forest cover depend on species traits?	Trzcinski et al. (1999), Newbold et al. (2013), Slade et al. (2013)
Does fragmentation increase community invasibility by promoting the spread of invasive species.	With (2004)
Questions on the importance of the matrix	
Does matrix habitat alter moderating impacts of dispersal on isolation distance between fragments?	Debinski (2006), Nichols et al. (2007)
How do matrix habitat and species traits interact in the response of biodiversity to forest fragmentation?	Kupfer et al. (2006), Kennedy et al. (2010), Prevedello and Vieira (2010)
Do cross-edge spillover effects of predators alter dynamics of prey populations in forest fragments (e.g., nest predation)?	Didham et al. (1996), Chalfoun et al. (2002), Rand et al. 2006

## Limitations of the Database

Whilst the BIOFRAG database represents an essential step toward improved analyses of biologic responses to fragmentation, it cannot directly address problems of suboptimal study design (Eigenbrod et al. 2011), data limitation (e.g., information not measured or excluded from response analyses) (Prugh et al. 2008), or varying data qualities produced by heterogeneous field measurements and unequal sampling effort. Also, varying species detectability may confound inference in meta-analyses and metrics calculated from aggregated data may be biased by sample size (Banks-Leite et al. 2012; Wells and O'Hara 2013). To raise awareness of these issues, the database includes for example information on sampling effort and measurement techniques. Details on the sampling technique, for example, measured attraction radius for light (Truxa and Fiedler 2012) or pitfall traps (Larsen and Forsyth 2005), and information on the use or nonuse of designed sampling protocols (Banks-Leite et al. 2012) is further examples of knowledge that could be included in the database.

## Concluding Remarks

Using the huge and valuable amount of primary data on biodiversity responses to fragmentation becomes increasingly important as anthropogenic pressures from burgeoning human populations and rising land demands are modifying landscapes, even in areas previously thought to be remote from human influence. Interstudy comparisons can aid in defining future research needs and in raising awareness of methodological inconsistencies, thereby paving the way for the design of standard, taxon-specific methods to measure responses to forest fragmentation. Collating fragmentation datasets from different eco-regions and realms provides the opportunity to develop our understanding of fragmentation derived from intensively sampled landscapes such as the BDFFP (Laurance et al. 2011), the Hope River forest fragmentation project (Ewers et al. 2002), and in the coming years from the Stability of Altered Forest Ecosystems Project (Ewers et al. 2011). The database places fragmentation as a focal issue in the broader context of land-use change and landscape level processes and highlights the continued need to move to landscape scale assessments. Output from the BIOFRAG database could be useful for online initiatives such as the Local Ecological Footprinting Tool (or LEFT) that uses global databases for assessing locally important ecological features of landscapes (e.g., beta-diversity, vulnerability, and fragmentation (Willis et al. 2012). This study is also a call to researchers to join the BIOFRAG community and share their data (given they meet the essential criteria, Tables S1 and S2, Fig. 1) with the BIOFRAG project and related research efforts such as PREDICTS. This will increase the capacity of the database to provide data for syntheses of land-use impacts on biodiversity at spatial resolutions relevant to critical decisions on future land allocations (Jetz et al. 2007; Platts 2012).

## Supporting Information

**Table S1.** Metadata of the BIOFRAG database.

**Table S2.** Information entered into the database.

**Figure S1.** Schematic structure of the BIOFRAG database.

**Figure S2.** Structure of the relational BIOFRAG database.
