# Supplementary material for: BIOFRAG – a new database for analyzing BIOdiversity responses to forest FRAGmentation
Source: Ecol Evol. 2014 Mar 27;4(9):1524–37. doi: 10.1002/ece3.1036 (PMC4063456; doi:10.1002/ece3.1036)
Supplement: Supplementary file 1 [file ece30004-1524-SD1.docx]

**S1 Table** Metadata of the BIOFRAG database. Clarification on any parts of the data set should be directed to Marion Pfeifer ([marion.pfeifer@gmail.com](mailto:d.eldridge@unsw.edu.au)).

| **1. Data set identity** | A relational global database that compiles primary biodiversity datasets from fragmented landscapes to analyse response(s) of one or more species linked to plots in fragments |
| --- | --- |
| **2. Responses** | Presence/absence data; (relative) abundance data; coverage data |
| **3. Data set name** | BIOFRAG_database.v1 |
| **4. Database** **Principal Investigators** | Marion Pfeifer, Department of Life Sciences, Imperial College London, Silwood Park Campus, Ascot, UK  Robert M Ewers, Department of Life Sciences, Imperial College London, Silwood Park Campus, Ascot, UK  Veronique Lefebvre, Department of Life Sciences, Imperial College London, Silwood Park Campus, Ascot, UK |
| **5. Database key words** | edge effects, forest fragmentation, forest degradation, landscape metrics, fragment traits, matrix contrast, species turnover, biodiversity turnover |
| **6. Format and storage mode** | Access database |
| **7. Missing or unavailable data** | Marked as 'NA' |
| **8. Study selection** | Essential criteria before inclusion of a dataset from these sources:   1. The datasets contain quantitative and therefore analysable data. 2. The datasets contain data on species responses that were measured in plots or along transect located within different fragments of a fragmented landscape 3. The datasets contain GPS coordinates and time-stamps for plots or transects sampled. If plots were measured repeatedly, the study has to specify whether data were stored separately for each sampling period or which aggregation techniques were applied to the response variables. 4. The datasets contain information on land cover type associated with each plot or transect. 5. The study identifies species to at least morphospecies level. 6. The study describes the method used to quantify response variables measured in the field. |
| **9. Data-use policy** | *Copyright or Proprietary Restrictions:* This data set is available for non-commercial scientific use, but researchers have to request access to individual datasets from the dataset authors. Meta-data are freely accessible and can be searched to identify the respective authors.  The database should be cited as: Pfeifer M et al. 2014. BIOFRAG – A new database for analysing BIOdiversity responses to forest FRAGmentation. Evology and Evolution. |
| **10. Website** | *http://biofrag.wordpress.com/about/* |

**S2 Table** Information entered into the database. See also Figure 1 in manuscript. Green: Data that are considered optional information.

| **Data provided by author of dataset** | |
| --- | --- |
| Spatial data | Plot Name |
|  | Latitude / Longitude |
|  | Habitat Type |
|  | Measurements: start date (day / month / year) |
|  | Altitude |
| Species data | Matrix of species abundances or presence/absence (or other trait) for each plot in Spatial Data |
|  | Genus and/or Species names |
| Methods (assumed equal across plots for a given inventory) | Measurement Technique (e.g. trap or sighting) |
|  | Number of measurements per plot (e.g. 1, 5 +, 10 +) |
|  | Technique used to aggregate measurements per plot (e.g. sum or average) |
|  | Spatial Accuracy (estimated confidence, e.g. on 0 to 5 scale) |
|  | Duration of measurements in plot (for how long) |
| Contact details | Name |
|  | Affiliation |
|  | Email |
| Publication record associated with inventory | Authors |
|  | Title |
|  | Year |
|  | Journal |
|  | Volume |
|  | Pages |
|  | |
| **Input optionally provided by author of dataset or derived by us** | |
| Spatial data | Country (current status) |
|  | Biogeographic Realm (WWF UNEP) |
|  | Biome (WWF UNEP) |
|  | Maps: source (e.g. satellite data used with time and day of recording and processing steps applied, projection, date, classification technique: e.g. supervised, legend) |
| Species data | Family / Order |
|  | Common / Alternative Name |
|  | Authority |
|  | IUCN conservation assessment status (Red List Status) |
| Seasonal Patterns | Type of season in the study area during measurement(s) |
| Habitats | IUCN habitat categories |


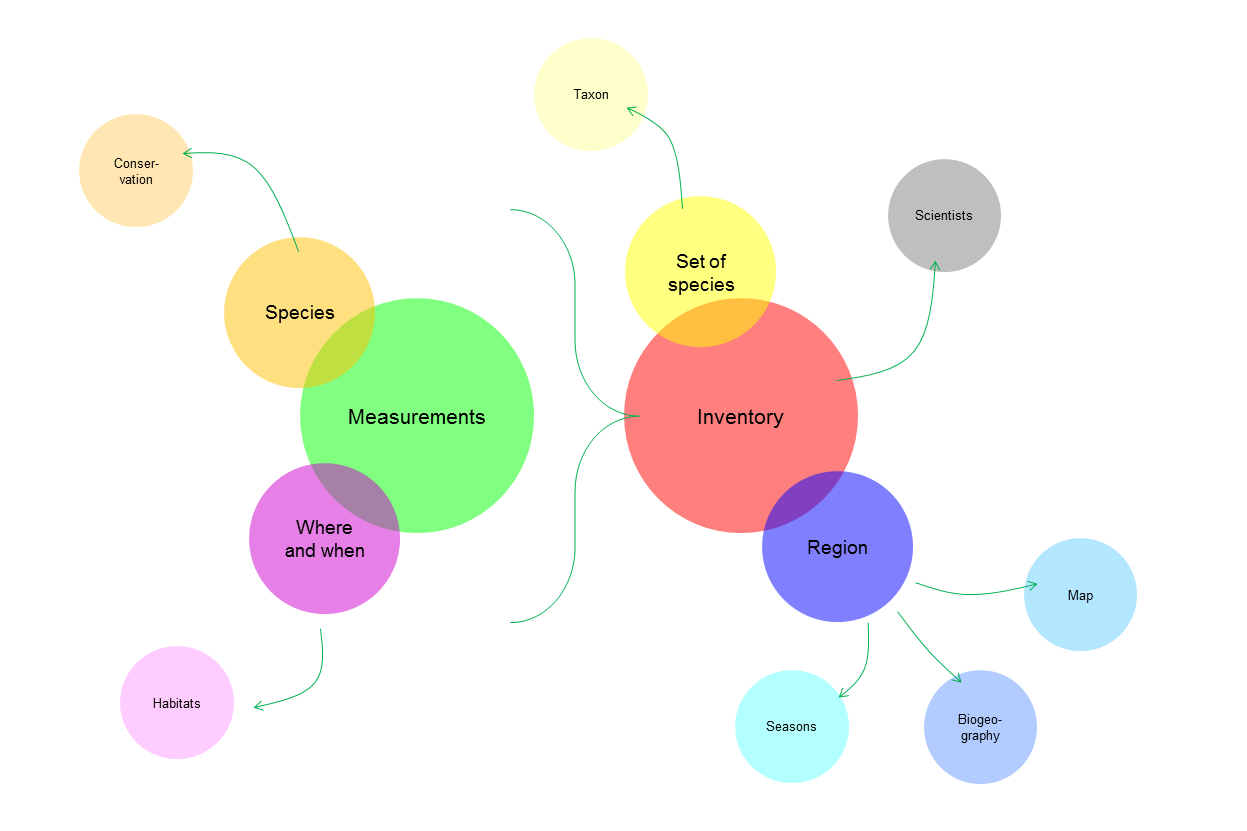


**S1 Figure** Schematic structure of the BIOFRAG database. The database is organised around six central tables. A record is for a particular species at a particular location and time. An inventory is a collection of records and therefore an association between a set of species and a set of locations in a time period. Each of these central tables is further defined by ‘outer’ tables. For example, a location and time refer to a habitat, whilst a set of locations and time refer to a country, a realm, or vegetation seasonality.

**
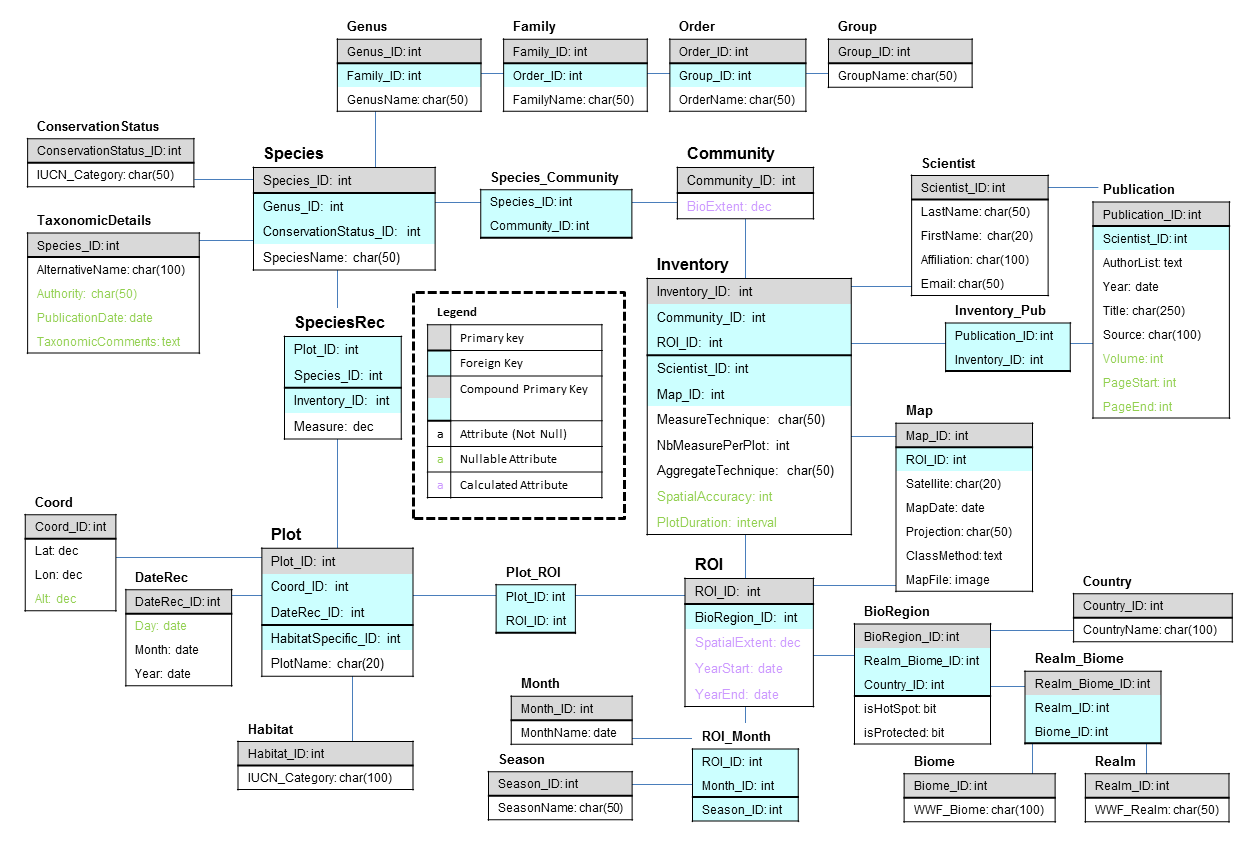
**

**S2 Figure** Structure of the relational BIOFRAG database. A primary key (grey fields) uniquely identifies each record (row) in a specific table. One or more attributes can be associated with each primary key in a specific table. A foreign key (light blue fields) links to a primary key in another table and is used to create relationships between tables. A primary key can be defined from the combination of foreign keys if it is unique in the table. Example: the combination of a month and a ROI is never repeated in the Month_ROI table. A primary key is not a row counter, if a record (row) is deleted, then its key is never assigned again. Tables with two foreign keys are called association tables. An attribute is a data field containing a single value of specific type (integer, decimal, char(lim), text, image..), pertaining to the table key only. Attributes should be independent of each other. Most attributes cannot be Null (data must be entered in these fields). The character string “unknown” (for instance for a conservation status) is not a Null. Only some optional attributes (such as altitude) may be Null. Some attributes are automatically calculated when the database is updated. For example, when an inventory is added the fields, YearStart and YearEnd of ROI are filled in by taking the minimum and maximum year across all the plots in the ROI. Similarly, aggregation operations can be performed for a set of species (community). Typical queries may be stored or performed automatically when updating the database (e.g. automated database statistics or data extraction (select map, sets of plots and sets of species) to perform ‘distance to edge’ analyses or ‘BIOFRAG Metric analyses). Database statistics can be queried by (1) counting the number of distinct rows (e.g. how many biomes, habitats, taxa, etc.), (2) using aggregation operations (e.g. min, max, mean: time span of records, species present per habitat, temporal variations of habitat at similar locations, list of habitats in a region, average number of datasets per author, set of publications for which an inventory contact appear in the author list, etc.). 1:n - The foreign key of the n table is added to the first table; n:n - A junction table is added combining the primary keys of two tables (i.e. two 1:n relations); 1:1 - Two tables sharing the same primary key.
